# Prevalence of QT prolongation and its risk factors in patients with type 2 diabetes

**DOI:** 10.1186/s12902-022-01235-9

**Published:** 2023-03-01

**Authors:** Khaled Aburisheh, Mohammad F. AlKheraiji, Saleh I. Alwalan, Arthur C. Isnani, Mohamed Rafiullah, Muhammad Mujammami, Assim A. Alfadda

**Affiliations:** 1 University Diabetes Center, King Saud University Medical City, King Saud University, Riyadh, Saudi Arabia; 2 Obesity Research Center, College of Medicine, King Saud University, P.O. Box 2925, Riyadh, 11461 Saudi Arabia; 3 Strategic Center for Diabetes Research, College of Medicine, King Saud University, Riyadh, Saudi Arabia; 4 Department of Medicine, College of Medicine, King Saud University, Riyadh, Saudi Arabia

**Keywords:** QTc prolongation, diabetes, ECG, QTc interval

## Abstract

**Background:**

QT prolongation increases cardiovascular mortality in diabetes. The risk factors for QT prolongation vary across different studies. There is no data on the QT prolongation in patients with diabetes from the Arab region, where diabetes is highly prevalent. Here we aimed to assess the prevalence of QT prolongation and its associated risk factors in patients with type 2 diabetes from Saudi Arabia.

**Method:**

This was a retrospective, cross-sectional, hospital-based file review study. Data were collected from the medical records of patients with type 2 diabetes aged above 14 years and underwent ECG examination, and laboratory investigations were done within one month of ECG.

**Results:**

The study included 782 patients with a prevalence of QTc prolongation of 13%. Patients with prolonged QTc interval were characterized by older age, higher BMI, longer diabetes duration, lower total cholesterol and LDL-C, and more diabetic nephropathy, hypertension, and CVD cases. They were also more in insulin treatment, antihypertensive medications, loop diuretics, and potassium-sparring diuretics. Logistic regression analysis revealed the odds of prolonged QTc interval increased significantly with CVD (OR = 1.761, 95% CI:1.021–3.036, *p* = 0.042), and usage of loop diuretics (OR = 2.245, 95% CI:1.023–4.923, *p* = 0.044) after adjusting for age, gender, and duration of diabetes.

**Conclusion:**

The risk factors associated with QTc prolongation in patients with type 2 diabetes are CVD, and loop diuretics. Age, BMI, and diabetes duration were more in people with QTc prolongation, whereas total cholesterol and LDL-C levels were lower. More patients had diabetic nephropathy, hypertension, and CVD with prolonged QTc.

**Supplementary Information:**

The online version contains supplementary material available at 10.1186/s12902-022-01235-9.

## Background

Diabetes-related mortality is mainly attributed to its cardiovascular complications [1]. Timely detection and treatment are essential in averting the high mortality associated with cardiovascular complications of diabetes. QT prolongation in the electrocardiogram (ECG) is one of the most commonly seen disorders of the heart. It may lead to potentially dangerous cardiac arrhythmia such as Torsade de Pointes. The QT interval represents the total duration of the ventricular depolarization and repolarization. Since the QT interval is influenced by the changes in the heart rate, a corrected QT interval (QTc) is used clinically [2]. It is usually found in patients with certain congenital conditions but can also be caused by several comorbid conditions and medications. Prolongation of QT interval is observed in many chronic inflammatory conditions, including diabetes [3]. Even though the effects of QT prolongation have been milder in the general population, it may increase the risk of mortality in patients with preexisting cardiac abnormalities. QTc prolongation independently predicted the cardiovascular mortality in people with diabetes [4]. Cardiovascular disease being one of the frequent complications associated with diabetes, the effect of QT prolongation in such patients need attention.

Patients with diabetes were reported to have a higher prevalence of QTc prolongation [5]. Patients with diabetes have a 2–10 times higher risk of sudden cardiac death than general population. Hyperglycemia and chronic changes in myocardium are the main factors behind the increased prevalence of QTc prolongation in patients with diabetes [6]. Hyperglycemia and coronary heart disease (CHD) were found to be the strong predictors of high QTc interval. In a population-based cross-sectional study, a high prevalence of QTc was observed in patients with diabetes. The CHD was independently associated with QTc interval prolongation even after adjustment for age and sex [7]. A recent study found that female gender and treatment with insulin sensitizers were the independent contributors for QTc interval prolongation [8]. QTc was found to independently predict all-cause mortality in a prospective cohort of people with type 2 diabetes [9]. Current HbA1c, long-term postprandial hyperglycemia, and higher glycemic variability in postprandial glucose levels were found to be strong independent risk factors of QTc prolongation [10].

Apart from hyperglycemia and cardiovascular disease (CVD), other diabetic complications and hypoglycemia were also found to be associated with QTc prolongation in patients with diabetes. Baseline QTc interval was significantly associated with the QTc prolongation during severe hypoglycemia in patients with type 2 diabetes [11]. QTc interval prolongation observed during hypoglycemia was independent of serum potassium levels [12]. Other metabolic diseases such as obesity, dyslipidemia, and hypertension were also found to be risk factors for QTc prolongation in patients with diabetes [13]. Although the association of QTc prolongation with various risk factors and diabetic complications has been reported in many studies, the results have mainly been inconsistent across studies. In addition, to our knowledge, there are no studies on the QTc prolongation in patients with diabetes from the Arab region, where diabetes is highly prevalent. Therefore, the present study is undertaken to assess the prevalence of QT prolongation and its associated risk factors in patients with type 2 diabetes from Saudi Arabia.

## Methods

This was a retrospective, cross-sectional, hospital-based file review study. The study population included patients with type 2 diabetes mellitus aged above 14 years who underwent ECG examination and laboratory investigations done within one month of the ECG recording. Patients with type 1 diabetes, pregnant women, patients using medications affecting QT interval (Supplementary Table 1), and patients with abnormal serum potassium levels were excluded (normal range 3.5–5 mmol/l). We did not exclude patients with atrial fibrillation or other arrhythmias. Patient records were screened from September 2013 to August 2015 using a simple random selection technique. The QT interval is defined as the time between the start of the Q wave and the end of the T wave in the heart’s electrical cycle. QTc was calculated by a 12-lead ECG machine automatically with Bazett formula (Esaote Biomedica Archimede Series 4200, Genova, Italy). The upper limit of normal was kept at 440 ms for males and 460 ms for females [14]. The study was conducted in accordance with the relevant guidelines and regulations and the study protocol was reviewed and approved by the Institutional Review Board, College of Medicine, King Saud University, Riyadh, Saudi Arabia.

The data was collected through file review based on a pre-designed data collection questionnaire. Medical records of all the patients were systematically reviewed to collect age, sex, body weight, height, body mass index (BMI), systolic and diastolic blood pressure (BP), heart rate, diabetes duration, presence of comorbidities such as hypertension and dyslipidemia, and presence of diabetic complications namely retinopathy, nephropathy, peripheral neuropathy, and vasculopathy. The laboratory data included fasting and two-hour postprandial blood glucose, glycated hemoglobin (HbA1c), and lipids (total cholesterol, high-density lipoprotein cholesterol (HDL-C), low-density lipoprotein cholesterol (LDL-C), and triglycerides). The details of medications prescribed to the patients were also collected. Diabetic nephropathy was considered if the patient had microalbuminuria (defined as a urinary albumin-to-creatinine level of 30–299 mg/g Cr) or macroalbuminuria (defined as a urinary albumin-to-creatinine level of more than 300 mg/g Cr). Diabetic neuropathy, vasculopathy, and retinopathy were considered positive if it was documented in the patients’ record. Retinopathy was reported when patients had either non-proliferative diabetic retinopathy or proliferative diabetic retinopathy with or without macular edema. The presence of at least one definite microaneurysm in any field of fundoscopy was considered as the diabetic retinopathy as assessed by an ophthalmologist. Neuropathy was considered based on physicians’ notes on the basis of neuropathic symptoms and signs or objectively abnormal results including insensitivity to a 10 g monofilament and abnormal vibration perception threshold based on the biothesiometer and without other significant disease.

### Statistical analysis

The data were analyzed using the Statistical Package for Social Sciences (SPSS) version 23.0 (IBM-SPSS, Armonk, New York, USA). Data are expressed as mean ± standard deviation for continuous variables and as number and percentage for categorical variables. Data were tested for normality of distribution. Significant differences in the mean were tested by the independent samples t-test. The chi-square test was used to determine whether there are statistically significant differences in the expected and observed frequencies in QTc prolongation (yes or no) according to gender, presence of comorbid conditions, family history of CVD, types of arrhythmia, PR interval, ST-segment results, and medications. The relationship between two continuous variables was tested using the Pearson’s correlation coefficient test. A multivariate logistic regression model was constructed for significant variables in the univariate and bivariate analyses associated with QTc prolongation. In the logistic regression model, QTc prolongation (dichotomized as prolonged and not prolonged) was the dependent variable, and significant covariates including BMI, LDL, total cholesterol, nephropathy, CVD, hypertension, use of insulin, loop diuretics, potassium-sparing diuretics and use of antiplatelet medication were included in the model as predictors for QTc prolongation. Logistic regression analysis for risk factors of prolonged QTc interval was done with adjustment for age, gender, and duration of diabetes. A *p*-value of ≤0.05 was considered statistically significant.

## Results

Data from 782 patients were included in the analysis, with 680 patients (87.0%) showing normal QTc intervals and 102 (13.0%) prolonged QTc intervals. The mean age of the study population was 56.4 years, and the mean duration of diabetes was 14.3 years. Retinopathy was the most frequent diabetic complication in our study population (41.2%), followed by neuropathy, nephropathy, and CVD. The majority of the included patients had dyslipidemia and hypertension (74.7 and 66.9%, respectively). Ninety-five percent of the patients were taking antidiabetic medications, with metformin being the most frequent antidiabetic treatment. The other frequently used drugs were meant for treating hypertension and dyslipidemia, followed by antiplatelet medications. The complete demographic, clinical characteristics, and medications of the study population are shown in Table 1.

**Table 1:** Demographic and clinical characteristics of the study population

Parameters	Mean ± SD or N (%)
Total number of patients	782
Age (years)	56.4+ ± 11.8
Sex	
Male	399 (51.0%)
Female	383 (49.0%)
Height (m)	161.2 ± 9.2
Body weight (kg)	82.9 ± 15.9
BMI (kg/m^2^)	31.9 ± 5.9
Duration of diabetes (years)	14.3 ± 8.7
HbA1c (%)	8.7 ± 1.8
Systolic BP (mmHg)	134 ± 16.9
Diastolic BP (mmHg)	74.4 ± 10.2
Heart rate (bpm)	83 ± 13.3
Fasting blood glucose (mm/L)	9.2 ± 3.5
Postprandial blood glucose (mmol/L)	13.4 ± 5.1
Total cholesterol (mmol/L)	4.4 ± 1.0
LDL-C (mmol/L)	2.5 ± 0.9
HDL-C (mmol/L)	1.23 ± 0.5
Triglycerides (mg/L)	1.6 ± 0.9
Retinopathy (%)	322 (41.2%)
Neuropathy (%)	273 (34.9%)
Nephropathy	159 (20.3%)
CVA	15 (1.9%)
CVD	121 (15.5%
PVD	27 (3.5%)
Hypertension	523 (66.9%)
Hyperlipidemia	584 (74.7%)
Sodium	138.4 ± 3.1
Potassium	4.2 ± 0.4
**Antidiabetic medications**	743 (95%)
Metformin	623 (79.7%)
Thiazolidinediones	124 (15.9%)
Sulfonylureas	279 (35.7%)
Meglitinides	36 (4.6%)
DDP-4 inhibitors	80 (10.2%)
Alpha-glucosidase inhibitors	39 (5%)
Insulin	322 (41.2%)
**Antihypertension medications**	448 (57.3%)
ACE inhibitors	114 (14.6%)
ARBs	254 (32.5%)
Calcium channel blockers	134 (17.1%)
Beta-blockers	126 (16.1%)
Thiazide diuretics	107 (13.7%)
Loop diuretics	35 (4.5%)
Potassium-sparing diuretics	4 (0.5%)
**Lipid lowering medications**	512 (65.5%)
Statins	494 (63.2%)
Fibrates	25 (3.2%)
Ezetimibe	5 (0.6%)
Anti-platelet medications	501 (64.1%)
Prolonged QTc	102 (13%)

*ACE inhibitors* Angiotensin-converting enzyme inhibitors, *ARB* Angiotensin receptor blocker, *BMI* Body mass index, *BP* Blood pressure, *CVA* Cerebrovascular accident, *CVD* Cardiovascular disease, *DPP-4 inhibitors* Dipeptidyl-peptidase 4 inhibitors, *HDL-C* High-density lipoprotein cholesterol, *LDL-C* Low-density lipoprotein cholesterol, *PVD* Peripheral vascular disease.

There was no significant difference in the gender distribution of the study population. The prevalence of QTc prolongation was not significantly different across gender (*p* = 0.135). Patients with prolonged QTc interval were significantly older (*p* = 0.001), had higher BMI (*p* = 0.030), longer diabetes duration (*p* = 0.050), and lower total cholesterol and LDL cholesterol levels (*p* = 0.019 and *p* = 0.017, respectively) compared to patients who had normal QTc interval. Diabetic nephropathy and CVD were the only diabetic complications that were significantly higher in patients with prolonged QTc intervals. Hypertension was more prevalent in patients with QTc prolongation. Comparing the diabetic medications, patients with prolonged QTc were significantly more insulin users. Moreover, antihypertensive and antiplatelet medications and diuretics (loop diuretics and potassium-sparing diuretics) were used more in patients with QTc prolongation (Table 2).

**Table 2:** Comparison of the patient characteristics between normal and prolonged QTc categories

Parameters	QTc prolonged ***N*** = 102	QTc normal ***N*** = 680	***p*** values
Age (years)	60.0 ± 11.0	55.8 ± 11.9	0.001*
Sex			
Male	45 (44.1%)	354 (52.1%)	0.135
Female	57 (55.9%)	326 (47.9%)	
Height (m)	161.1 ± 8.6	161.3 ± 9.3	0.831
Body weight (kg)	85.5 ± 13.9	82.5 ± 16.1	0.070
BMI (kg/m^2^)	33.1 ± 5.4	31.8 ± 6.0	0.030*
Duration of diabetes (years)	15.9 ± 9.0	14.1 ± 8.6	0.050
HbA1c (%)	8.6 ± 1.7	8.7 ± 1.8	0.565
Systolic BP (mmHg)	135.1 ± 17.4	133.9 ± 16.9	0.509
Diastolic BP (mmHg)	73.6 ± 9.8	74.5 ± 10.3	0.434
Heart rate (bpm)	83.4 ± 14.9	82.9 ± 13.1	0.723
Fasting blood glucose (mm/L)	9.1 ± 3.5	9.2 ± 3.5	0.790
Postprandial blood glucose (mmol/L)	13.5 ± 4.4	13.4 ± 5.1	0.922
Total cholesterol (mmol/L)	4.2 ± 1.0	4.4 ± 1.0	0.019*
LDL-C (mmol/L)	2.3 ± 0.8	2.5 ± 0.9	0.017*
HDL-C (mmol/L)	1.23 ± 0.6	1.23 ± 0.5	0.928
Triglycerides (mg/L)	1.6 ± 0.8	1.7 ± 0.9	0.590
Retinopathy (%)	45 (44.1%)	277 (40.7%)	0.517
Neuropathy (%)	37 (36.3%)	236 (34.7%)	0.757
Nephropathy	29 (28.4%)	130 (19.1%)	0.029
CVA	2 (2.0%)	13 (1.9%)	0.973
CVD	29 (28.4%)	92 (13.5%)	< 0.001*
PVD	2 (2.0%)	25 (3.7%)	0.376
Hypertension	78 (76.5%)	445 (65.4%)	0.027*
Hyperlipidemia	81 (79.4%)	503 (74.0%)	0.239
Sodium	138.4 ± 3.1	138.4 ± 3.1	0.799
Potassium	4.2 ± 0.4	4.2 ± 0.4	0.780
**Diabetes medications**	98 (99.0%)	645 (99.8%)	0.126
Sensitizers	78 (76.5%)	545 (80.1%)	0.390
Thiazolidinediones	12 (11.8%)	112 (16.5%)	0.225
Secretagogues	32 (21.4%)	247 (36.3%)	0.330
Meglitinides	3 (2.9%)	33 (4.9%)	0.390
DDP4 inhibitors	17 (13.7%)	66 (9.7%)	0.212
Alphaglucosidase inhibitors	7 (6.9%)	32 (4.7%)	0.351
Insulin	53 (52.0%)	269 (39.6%)	0.018*
**Anti-hypertension medications**	72 (70.6%)	376 (55.3%)	0.004*
ACE inhibitors	19 (18.6%)	959 (14.0%)	0.214
ARBs	33 (32.4%)	221 (32.5%)	0.976
Calcium channel blockers	24 (23.5%)	110 (16.2%)	0.066
Beta-blockers	22 (21.6%)	104 (15.3%)	0.108
Thiazide diuretics	15 (14.7%)	92 (13.5%)	0.747
Loop diuretics	14 (13.7%)	21 (3.1%)	< 0.001*
Potassium-sparing diuretics	2 (2.0%)	2 (0.3%)	0.028*
**Lipid lowering medications**	71 (69.6%)	441 (64.9%)	0.346
Statins	71 (69.6%)	423 (62.2%)	0.148
Fibrates	3 (2.9%)	22 (3.2%)	0.875
Ezetimibe	1 (1.0%)	4 (0.6%)	0.643
Anti-platelet medication	75 (73.5%)	426 (62.6%)	0.033*

*ACE inhibitors* Angiotensin-converting enzyme inhibitors, *ARB* Angiotensin receptor blocker, *BMI* Body mass index, *BP* Blood pressure, *CVA* Cerebrovascular accident, *CVD* Cardiovascular disease, *DPP-4 inhibitors* Dipeptidyl-peptidase 4 inhibitors, *HDL-C* High-density lipoprotein cholesterol, *LDL-C* Low-density lipoprotein cholesterol, *PVD* Peripheral vascular disease

*p ≤ 0.05

A logistic regression model was constructed with QTc prolongation as the dependent variable and significant factors including BMI, total cholesterol, LDL-C, nephropathy, CVD, hypertension, and the use of insulin, loop diuretics, potassium-sparing diuretics, and antiplatelet medication as independent factors with adjustment for age, gender, and duration of diabetes. The logistic regression analysis showed the following significant predictors for prolonged QTc interval; the odds of prolonged QTc interval increased significantly with CVD (OR = 1.761, 95%CI:1.021–3.036, *p* = 0.042), and usage of loop diuretics (OR = 2.245, 95%CI:1.023–4.923, *p* = 0.044) (Table 3).

**Table 3:** Logistic regression analysis for predictors of prolonged QTc interval

Predictors	B	OR (ExpB)	95% CI	***P*** value^**$**^
Nephropathy	0.251	1.285	0.766–2.158	0.342
CVD	0.556	1.761	1.021–3.036	0.042*
Hypertension	−0.190	0.827	0.482–1.419	0.490
BMI	0.025	1.025	0.988–1.064	0.188
LDL-C	0.016	1.016	0.656–1.574	0.944
Total cholesterol	−0.163	0.849	0.581–1.241	0.398
Insulin	0.271	1.312	0.590–2.917	0.506
Anti-platelet medications	0.254	1.289	0.789–2.108	0.311
Loop diuretics	0.809	2.245	1.023–4.923	0.044*
Potassium-sparing diuretics	1.047	2.849	0.345–23.518	0.331

*BMI* Body mass index, *CVD* Cardiovascular disease, *LDL-C* Low-density lipoprotein cholesterol

^$^Adjusted for age, gender, and duration of diabetes

*p ≤ 0.05

## Discussion

There is wide variability in the reported prevalence of QTc prolongation in the diabetic population ranging from 11.3 to 59.3% [15, 16]. It might be a consequence of the heterogeneity in the definition of prolonged QTc and the differences in the mean age of the populations. Moreover, inaccurate identification of the beginning and the end of the QT interval by the observer or the software in the automatic analysis of ECG tracings could also be behind this variation. The prevalence of the prolonged QTc in our study was 13%. Even though a higher proportion of women showed QTc prolongation, it was not statistically significant. Female gender was reported to be an independent risk factor for QTc prolongation in patients with diabetes in a cross-sectional cohort study where a different criterion for prolonged QTc in females was not used [17]. Despite a higher cut-off to define the QTc prolongation in females in our study, the prevalence of QTc prolongation in females was only slightly higher.

The results show that patients with QTc prolongation were older in our study. Similar results were seen in the diabetic and general population [16, 17]. Older people are more likely to have worsening diabetes and complications, especially CHD, which could predispose them to alterations in the QTc interval. Patients with increased QTc interval were found to have a higher BMI This is in confirmation with a previously reported study [17]. BMI is a known risk factor for cardiovascular diseases and is also associated with a poor prognosis of diabetes. However, in another study, there was no difference in the BMI between patients with prolonged QTc and normal QTc intervals [7]. Notably, the duration of diabetes was similar between the patient groups in these studies, whereas in our study, the patients with prolonged QTc had a slightly longer duration.

The effect of glycemia on the QT prolongation has not been consistent. Ninkovic et al. reported that most of the glycemic parameters like fasting blood glucose, postprandial blood glucose, HbA1c, mean blood glucose, and mean glucose excursions were significantly higher in individuals with QTc prolongation [17]. In addition, mean blood glucose was also found to be predictive of prolonged QTc interval and QTc dispersion. However, in another study, no differences were found in the HbA1c between normal and prolonged QTc interval groups [7]. In Diabetes Cohort Study, the baseline blood glucose and HbA1c levels were similar between patients with prolonged QTc interval and normal QTc interval [9]. The present study found no difference in fasting and postprandial blood glucose and HbA1c levels between the study groups.

The most common comorbidities of diabetes like hypertension and dyslipidemia were associated with a higher prevalence of QT prolongation in individuals with diabetes [7, 13]. Both systolic and diastolic pressures were found to be significantly higher in people with QTc prolongation. Our results show that cases of hypertension were significantly higher in individuals with prolonged QTc. The systolic and diastolic blood pressures were not different between the two groups. As the patients with QTc prolongation had significantly higher usage of antihypertensive medications, the observation of comparable blood pressure levels between the groups is understandable. Many studies suggest that the association of hypertension with left ventricular hypertrophy induced changes in the myocardium and sympathovagal imbalance as likely mechanisms behind the relationship of hypertension and QTc prolongation [7, 18]. Previous studies reported either higher cholesterol levels in patients with QTc prolongation or no difference [9, 13]. Interestingly, our results show that patients with QTc prolongation were characterized by lower total cholesterol and LDL-C levels. With the high prevalence of CVD in patients with prolonged QTc, those patients might likely have been on intensive statin therapy. However, it is not clear whether this would have been large enough to produce a significant difference in the lipid profile.

Diabetic nephropathy and CVD are the complications that were present significantly higher in patients with prolonged QTc intervals in our study. Previous studies reported more patients with prolongation of QTc intervals had diabetic retinopathy, neuropathy, and nephropathy [17, 19]. The presence of microalbuminuria is known to prolong QTc interval in individuals with type 2 diabetes. However, the pathophysiology behind the coexistence of both conditions is not understood. Independent association of diabetic complications such as neuropathy and nephropathy with QTc prolongation was reported previously [19]. But, in our study, we found CVD, the only diabetic complication associated independently with prolonged QTc interval. Coronary ischemia, infarction, and other cardiac abnormalities can induce QTc prolongation [20]. Ischemia causes structural damage of the heart and is associated with hyperactivity of the sympathetic system, leading to an increase in the ventricular depolarization duration represented by the QT interval in the ECG [17].

In our study population, insulin treatment was higher among patients with QTc prolongation. A similar observation was found by Kobayashi et al. [19]. The influence of insulin on the QTc interval can be in many ways. Patients on insulin treatment are likely to be older with a longer duration of diabetes and uncontrolled diabetes. These factors are known to be associated with the prolongation of QTc. In addition, insulin is likely to cause QTc prolongation by altering the sympathetic activity and serum potassium concentration [21]. Insulin-induced hypoglycemia can also cause a prolongation of QTc intervals [22]. Despite being involved in multiple mechanisms, the use of insulin did not significantly increase the risk of QTc interval prolongation. We found loop diuretics as independent predictors of QTc prolongation. These drugs were reported to be predictors of QTc prolongation previously [23]. Usually, loop diuretics do not cause QTc interval prolongation, and their effect on QTc prolongation was independent of electrolyte changes [24]. But since the patients treated with loop diuretics were likely to have hypertension and cardiovascular disease, more patients treated with loop diuretics likely had QTc prolongation. Potassium-sparring diuretics were linked to QTc interval shortening, and it was dependent on the serum electrolytes levels [24]. Our study population had only a few patients using potassium-sparing diuretics, and we excluded patients with abnormal serum potassium levels. Therefore, the results need to be interpreted cautiously. Intake of antiplatelet medications was seen higher in patients with prolonged QTc intervals. Aspirin and clopidogrel were the antiplatelet medications used by our study patients. These drugs are usually prescribed to patients with diabetes who have additional risk factors for cardiovascular diseases or as a secondary prevention strategy in patients with preexisting cardiovascular disease. Our study population has significantly more patients having cardiovascular disease and prolonged QTc. Therefore, people with QTc prolongation are more likely to have had antiplatelet medications. In addition, clopidogrel is associated with prolongation of QTc interval [25]. Since fewer patients were on clopidogrel in our study, its influence on the overall outcome is likely to be negligible.

The present study has identified the risk factors associated with QTc prolongation in patients with type 2 diabetes. CVD, and diuretic medications (loop diuretics) were significantly associated with prolonged QTc interval after adjusting age, gender, and duration of diabetes. The patient population with prolonged QTc interval was characterized by older age, higher BMI, longer duration of diabetes, lower total cholesterol and LDL-C, and more diabetic nephropathy, hypertension, and CVD cases. The number of patients with prolonged QTc was also more under insulin treatment, antihypertensive medications, and diuretics (loop diuretics). While most of these factors could be explained for their link with QTc prolongation, lower total cholesterol and LDL-C levels need further investigation. It is not clear whether it was an outcome of intensive statin therapy in patients with QTc prolongation. Our study has several limitations. This is a retrospective cross-sectional study; therefore, the findings are not suggestive of temporal or causal relationships between risk factors and QTc interval prolongation. Since our study is based on file documentation review, it could have led to biased data analysis. Further, we did not analyze the other QT parameters, such as QT dispersion which has been shown to have a predictive role in all-cause and cardiovascular mortality in type 2 diabetes.

## Conclusion

The risk factors associated with QTc prolongation in patients with type 2 diabetes were found to be CVD, and diuretic medications (loop diuretics). Age, BMI, and duration of diabetes were more in people with QTc prolongation, whereas total cholesterol and LDL-C levels were lower. More patients had diabetic nephropathy, hypertension, and CVD in individuals with prolonged QTc.

## Supplementary Information

**Additional file 1:** **Supplementary Table 1.** List of excluded medications known to affect the QTc interval.

## Data Availability

The datasets generated and/or analysed during the current study are not publicly available due local regulations but are available from the corresponding author on reasonable request.

## Abbreviations

ACE inhibitors: Angiotensin-converting enzyme inhibitors
ARB: Angiotensin receptor blocker
BMI: Body mass index
BP: Blood pressure
CHD: Coronary heart disease
CVA: Cerebrovascular accident
CVD: Cardiovascular disease
DPP-4 inhibitors: Dipeptidyl-peptidase 4 inhibitors
ECG: Electrocardiogram
HbA1c: Glycated hemoglobin
HDL-C: High-density lipoprotein cholesterol
LDL-C: Low-density lipoprotein cholesterol
OR: Odds ratio
PVD: Peripheral vascular disease
QTc: corrected QT interval
