# Supplementary material for: Prevalence of QT prolongation and its risk factors in patients with type 2 diabetes
Source: BMC Endocr Disord. 2023 Mar 1;23:50. doi: 10.1186/s12902-022-01235-9 (PMC9976503; doi:10.1186/s12902-022-01235-9)
Supplement: Supplementary file 1 — Additional file 1: Supplementary Table 1. List of excluded medications known to affect the QTc interval. [file 12902_2022_1235_MOESM1_ESM.docx]

**Supplementary Table 1. List of excluded medications known to affect the QTc interval**

| **Category** | **Medication** |
| --- | --- |
| Antibiotics |  |
|  | Azithromycin |
|  | Clarithromycin |
|  | Erythromycin |
|  | Roxithromycin |
|  | Moxifloxacin |
| Antifungals |  |
|  | Fluconazole |
|  | Ketoconazole |
| Antimalarials |  |
|  | Chloroquine |
|  | Mefloquine |
| Antiarrhythmics |  |
|  | Disopyramide |
|  | Procainamide |
|  | Quinidine |
|  | Amiodarone |
|  | Sotalol |
| Tricyclic Antidepressants |  |
|  | Amitriptyline |
|  | Clomipramine |
|  | Imipramine |
|  | Dothiepin |
|  | Doxepin |
| Antipsychotics |  |
|  | Risperidone |
|  | Fluphenazine |
|  | Haloperidol |
|  | Clozapine |
|  | Thioridazine |
|  | Ziprasidone |
|  | Pimozide |
|  | Droperidol |
| Antihistamines |  |
|  | Terfenadine |
|  | Astemizole |
| Others |  |
|  | Probucol |
|  | Cisapride |
|  | Nelfinavir |
